# Aberrated Multidimensional EEG Characteristics in Patients with Generalized Anxiety Disorder: A Machine-Learning Based Analysis Framework

**DOI:** 10.3390/s22145420

**Published:** 2022-07-20

**Authors:** Zhongxia Shen, Gang Li, Jiaqi Fang, Hongyang Zhong, Jie Wang, Yu Sun, Xinhua Shen

**Affiliations:** 1 School of Medicine, Southeast University, Nanjing 210096, China; snowszx@sina.com; 2 Sleep Medical Center, Huzhou Third Municipal Hospital, The Affiliated Hospital of Huzhou University, Huzhou 313000, China; 3 College of Mathematical Medicine, Zhejiang Normal University, Jinhua 321017, China; fjq0971@zjnu.edu.cn (J.F.); zhonghongyang@zjnu.edu.cn (H.Z.); zjnuwj@zjnu.edu.cn (J.W.); 4 Key Laboratory for Biomedical Engineering of Ministry of Education of China, Department of Biomedical Engineering, Zhejiang University, Hangzhou 310027, China; yusun@zju.edu.cn

**Keywords:** generalized anxiety disorder (GAD), electroencephalogram (EEG), functional connectivity (FC), fuzzy entropy (FE), power spectrum density (PSD), machine learning

## Abstract

Although increasing evidences support the notion that psychiatric disorders are associated with abnormal communication between brain regions, scattered studies have investigated brain electrophysiological disconnectivity of patients with generalized anxiety disorder (GAD). To this end, this study intends to develop an analysis framework for automatic GAD detection through incorporating multidimensional EEG feature extraction and machine learning techniques. Specifically, resting-state EEG signals with a duration of 10 min were obtained from 45 patients with GAD and 36 healthy controls (HC). Then, an analysis framework of multidimensional EEG characteristics (including univariate power spectral density (PSD) and fuzzy entropy (FE), and multivariate functional connectivity (FC), which can decode the EEG information from three different dimensions) were introduced for extracting aberrated multidimensional EEG features via statistical inter-group comparisons. These aberrated features were subsequently fused and fed into three previously validated machine learning methods to evaluate classification performance for automatic patient detection. We showed that patients exhibited a significant increase in beta rhythm and decrease in alpha1 rhythm of PSD, together with the reduced long-range FC between frontal and other brain areas in all frequency bands. Moreover, these aberrated features contributed to a very good classification performance with 97.83 ± 0.40% of accuracy, 97.55 ± 0.31% of sensitivity, 97.78 ± 0.36% of specificity, and 97.95 ± 0.17% of F1. These findings corroborate previous hypothesis of disconnectivity in psychiatric disorders and further shed light on distribution patterns of aberrant spatio-spectral EEG characteristics, which may lead to potential application of automatic diagnosis of GAD.

## 1. Introduction

Generalized anxiety disorder (GAD), a subtype of the anxiety disorder, is characterized by frequent/persistent, generalized feelings of nervousness and excessive anxiety without a clear object or fixed content [1]. It often manifests as overmuch worriedness or annoyance about real-life problems [2] (i.e., worrying about work, illness, financial situations, social competence, or accidents of oneself or relatives), leading to undesired and inappropriate feelings for the patients that cannot be easily off-loaded. According to the previous reports, the prevalence of the GAD is up to 3.1% every year or 5.7% over the lifetime in the United States of America (USA) [3], while the prevalence of GAD in urban China is 5.3% [4]. Hence, it has attracted growing attention in research due to the high prevalence rate as well as its common-existence in our daily surroundings [5,6]. Specifically, recent findings showed that GAD usually appears during mid-adolescence [7,8] and can be unremitting throughout life if not treated properly [6]. Despite recent advancements in the understanding of the physiological and psychological of GAD, the electrophysiological correlates of GAD still remain poorly understood [9].

Electroencephalogram (EEG) is an important non-invasive and economical electrophysiological measurement technique that explores neuronal activity by placing electrodes on the scalp [10]. EEG has a high temporal resolution, on the same millisecond scale as brain neural activity (synaptic transmission is on the order of 1 millisecond, and information transmission is on the order of hundreds of milliseconds), enabling accurate acquisition of neural activity and characterization of functional changes during cerebral dynamic activity [11]. Up to now, EEG has been widely utilized in revealing the etiology of various mental disorders (i.e., anxiety [12], depression [13], epilepsy [14], schizophrenia [15], Alzheimer [16], Parkinson [17], Autism Spectrum Disorder [18], etc.) due to its low-cost, easy-to-use, and flexible regions-of-interest configuration [19,20]. Of note, power spectrum density (PSD) analysis (which is a common power analysis method, mainly including power, relative power, power ratio, power spectral coupling, etc.) with typical EEG rhythms showed GAD related aberrations. For instance, Oathes et al. reported that patients with GAD exhibited high power in the gamma band in posterior channels that have long been recognized to be associated with negative emotion [21]. More recently, the power spectral coupling between the delta and beta oscillations has been proven to be related to GAD [22].

Although researchers have become increasingly interested in studying normal and pathological brain functions via the interactions between different brain regions [22,23], very few investigations of EEG functional connectivity (FC) have been performed on GAD recently. FC in brain neuroscience represents the correlation of simultaneous activity between different brain anatomical areas, and is essentially a statistical connotation that captures statistically dependent changes among the distributed remote neural ensembles [24]. According to the global neuronal workspace theory, the brain relies on multiple functional brain regions even performing very simple tasks, and the changes of the brain functions will inevitably map onto the FCs between the entire functional brain regions. Therefore, FC analysis can provide a unique quantitative approach to analyze the neural mechanism of GAD from the perspective of whole brain functional network. From the network topology perspective, it has been found that theta rhythm network integration (measured by characteristic path length) increased as the cognitive load increased during more complex emotion regulation tasks in patients with GAD [25]. EEG-based functional connectome is highly dynamic and specific, and it can serve as a promising non-invasive biomarker for GAD diagnostic.

In this study, we expanded these early studies in regard of proposing a novel analysis framework incorporating multidimensional EEG characteristics extraction and machine learning analysis, with an ultimate aim of augmenting our understanding of the underlying aberrant neural mechanisms of GAD and automatic GAD detection. Specifically, the nascent filed of FC studies of GAD [9] was adopted together with the previously validated univariate EEG studies (i.e., PSD and entropy [26]) for multidimensional EEG characteristics construction, which were then subjected to a conventional feature selection approach via inter-group statistical comparisons. Then the statistically different features were used as input for three widely utilized machine learning techniques. We expect to reveal neurobiological markers via the proposed analytical framework. In addition, among the existed studies [26,27,28], the highest classification accuracy between GAD and HC group, to our knowledge, is 93% with a deep learning model using task EEG signals. We also hope the proposed analysis framework could provide an effective and reliable GAD identification method to achieve better classification performance with machine learning models.

## 2. Materials and Methods

### 2.1. Participants

Forty-five patients who met the Diagnostic and Statistical Manual of Mental Disorders-IV (DSM-IV) criteria for GAD were recruited from Huzhou Third People’s Hospital, and thirty-six healthy controls (HC) were recruited from the local community and assessed by a psychiatrist using the Structural Clinical Interview for DSM-IV Disorders. The demographic and clinical characteristics of the participants are given in Table 1. As shown in Table 1, the mean age of GAD was 41.8 ± 9.4 (13 males and 32 females), and the mean age of HC was 36.9 ± 11.3 (11 males and 25 females), with no statistical differences between GAD and HC. All subjects should participate in two questionnaires, Hamilton Rating Scale for Anxiety (HAMA) scores and 17-item Hamilton Rating Scale for Depression (HAMD-17), and meet the following requirements: HAMA scores ≥ 17 and HAM-D17 ≤ 14 for GAD; HAMA scores ≤ 7 and HAMD-17 ≤ 7 for HC. In addition, each participant should be right-handed, no other mental disorders (such as dementia, schizophrenia, epilepsy, delusional disorder, bipolar disorder, depression disorder and so on, except GAD) and physical disorders (such as severe cardiopulmonary, hepatorenal insufficiency, malignant tumor or hematopathy, autoimmune diseases and so on) that may impair brain functions, no substance and alcohol abuse, and no signs of brain damage, which were determined by self-reported. Each participant was also required to not stay up late, not drink alcohol and drugs within one day before the experiment, and no smoking, coffee and tea in 8 h before EEG recording. The experiment was permitted by the Ethics Committee of Huzhou Third Municipal Hospital. Written informed consent was obtained from all participants before the test.

### 2.2. EEG Data Acquisition and Preprocessing

Sixteen-channel EEG signals, Fp1, Fp2, F3, F4, C3, C4, P3, P4, O1, O2, F7, F8, T7, T8, P7, and P8, were collected by an EEG apparatus (Nicolet EEG TS215605) according to the international 10–20 system, and referenced to the average of the left and right mastoids. The sampling rate was 250 Hz and the electrode impedance was controlled below 5000 Ω. Each subject was required to close their eyes, be awake and relaxed for ten minutes to collect 10- m continuous EEG data. The whole experiment was implemented in a professional EEG lab in Huzhou Third People’s Hospital.

A popular preprocessing procedure was applied for the EEG data. Firstly, the raw EEG data were down sampled from 250 Hz to 100 Hz, and filtered between 4 Hz and 30 Hz by a digital pass filter of fourth-order Butterworth band. Then, fast ICA was utilized for artifacts removal (such as eye blinks, slow eye movements and so on). Next, 4 s of continuous EEG data with 50% overlap were singled out as an EEG sample, resulting in 10,273 samples for GAD group and 7773 samples for HC group. It is important to note that all the further analyses are based on 4-s continuous EEG data. Finally, EEG rhythms of theta (4–8 Hz), alpha1 (8–10 Hz), alpha2 (10–13 Hz), and beta (13–30 Hz) were extracted for every EEG sample by the same band pass filter.

### 2.3. EEG Features Extraction

Numerous studies have demonstrated the feasibility of utilizing EEG features to explore and detect mental disorders. As mentioned previously, the primary objective of the current work is to develop a feasible and efficient method for automatic GAD detection via utilizing multidimensional EEG characteristics. To this end, three widely used EEG features (including PSD analysis, fuzzy entropy (FE) analysis, and FC analysis), which have been repeatedly demonstrated their effectiveness in detecting mental disorders [29], were adopted in this work. These three methods decode the information contained in EEG signals from three different dimensions, which portrayed the neurophysiological implications of EEG signals from three different perspectives. They are very representative. Specifically, PSD, FE, and phase lag index (PLI, which is a common method for determining the weights of FCs) are calculated for each EEG sample.

#### 2.3.1. PSD Calculation

For the given EEG signal *x*(*i*) (*i* = 1, 2, 3, …, *N*; *N* is the number point of *x*(*i*)), its frequency spectrum *X*(*f*) can be estimated by fast Fourier transform (FFT), and then the power spectrum *P_x_*(*f*) was gained with Equation (1). The relative power of *PSD*(*h*) for each EEG rhythm can be computed through Equation (2). In Equation (2), *h* represents the EEG rhythms of theta, alpha1, alpha2, and beta, *f_m_* and *f_n_* are the upper and lower frequencies of the EEG signal with four rhythms, while *f_h_* and *f_l_* are the upper and lower frequencies of *h* rhythm, respectively.

(1) $$P_{x}(f)=\frac{1}{N}{\left|X(f)\right|}^{2}$$

(2) $$PSD\left(h\right)=\frac{\int_{f_{l}}^{f_{h}}P_{x}\left(f\right)df}{\int_{f_{n}}^{f_{m}}P_{x}\left(f\right)df}\times \frac{f_{m}-f_{n}}{f_{h}-f_{l}}$$

#### 2.3.2. FE Calculation

For the given EEG signal *x*(*i*) (*i* = 1, 2, 3, …, *N*), it can be reconstructed into a set of *m*-dimensional vectors $X_{i}^{m}$ shown in Equation (3), where *m* is the embedding dimension, and *x*_0_(*i*) represents the mean value shown in Equation (4). The distance $d_{ij}^{m}$ between $X_{i}^{m}$ and $X_{j}^{m}$ is calculated by Equation (5). Then the similarity $D_{ij}^{m}$ between $X_{I}^{m}$ and $X_{j}^{m}$ is defined as Equation (6). The *O^m^*(*r*) is calculated by Equation (7). The FE [30] of the EEG signal *x*(*i*) could then be estimated as Equation (8).

(3) $$X_{i}^{m}=\{x\left(i\right),x\left(i+1\right),\cdots x\left(i+m-1\right)\}-x_{0}\left(i\right),\left(i=1,2,\cdots ,N-m+1\right)$$

(4) $$x_{0}(i)=\frac{1}{m}\sum_{j=0}^{m-1}u(i+j)$$

(5) $$d_{ij}^{m}=\underset{k\in (1,m)}{\max}\left\{\left|\left(u(i+k)-u_{0}(i))-(u(j+k)-u_{0}(j)\right)\right|\right\},(i\neq j)$$

(6) $$D_{ij}^{m}=\exp(-\ln(2)\times \left(\frac{d_{ij}^{m}}{r}{)}^{2}\right)$$

(7) $$O^{m}(r)=\frac{1}{N-m}\sum_{i=1}^{N-m}\left(\frac{1}{N-m-1}\sum_{j=1,j\neq i}^{N-m}D_{ij}^{m}\right)$$

(8) $$FE(m,r,N)=\ln O^{m}(r)-\ln O^{m+1}(r)$$

In the current study, a typical value for the embedding dimension *m* is set as 2, and the value *r* is determined by *k* × *δ*, *N* is the length of the EEG signal *x*(*i*) under observation (*N* = 1000). Additionally, *k* is the constant value set as 0.2 (usually the value range is between 0.10 and 0.25), and *δ* is the standard deviation of the EEG signal *x*(*i*).

#### 2.3.3. PLI Calculation

For the given EEG time series *x_i_*(*t*), it can be expressed as *z_i_*(*t*) by Hilbert transform [31] shown in Equation (9):

(9) $$z_{i}(t)=Z_{i}(t)e^{j\varphi_{i}(t)}$$

where *Z_i_* and *φ_i_* are the instantaneous amplitude and phase of *x_i_*(*t*), respectively. Then the PLI [32] of two EEG time series *x_k_*(*t*) and *x_l_*(*t*) can be estimated by Equation (10):

(10) $$PLI_{k,l}=\left|\langle sign(\varphi_{k}(t)-\varphi_{l}(t))\rangle \right|$$

where *sign* is sign function, <•> means the average value, and |•| denotes the absolute value. The PLI value ranges between 0 and 1. PLI = 0 represents the case where there is no phase synchronization, while PLI = 1 indicates the perfect phase locking between two EEG time series.

### 2.4. Feature Selection via Statistical Analysis

Once the multidimensional EEG features were constructed, a feature selection approach was needed given that the number of features is relatively large (i.e., PSD, 16 EEG channels × 4 frequency bands; FE, 16 EEG channels × 4 frequency bands; PLI, 16 × (16 − 1)/2 functional connections × 4 frequency bands) that may contain irrelevant features and redundant features. Here, a conventional statistical comparison approach was used for feature section. Specifically, a separate one-way analysis of variance (ANOVA) was carried out to determine the significant statistical differences of EEG characteristics between GAD and HC groups. Of note, this one-way ANOVA was performed on the PSD, FE, and FCs. The statistical differences were considered significant at a threshold of 0.05 (*p* < 0.05).

### 2.5. Machine Learning for Classification

In this study, three previously validated machine learning models [29], support vector machine (SVM), random forest (RF), and ensemble learning of back propagation neural network based on bagging strategy (BP_Bagging), were utilized with 10 times of hold-out method for cross-validation (80% samples for training and 20% samples for testing), respectively. Specifically, the kernel function of radial basis function (RBF) was applied for SVM, and 500 decision trees were utilized for RF. As for BP_Bagging, 100 base learners with 6 hidden layers and 100 neuron cells were used for ensemble, 80% of features and train samples were implemented for feature perturbation and sample perturbation, and then the voting method was performed on the outputs of 100 base learners to gain the final classified result. The performances of the three models are evaluated by accuracy [(TP + TN)/(TP + TN + FP + FN)], sensitivity [TP/(TP + FN)], specificity [TN/(FP + TN)], F1 [2TP/(2TP + FP + FN)]. Where, TP is true positive, FP is false positive, FN is false negative, and TN is true positive. Besides, all analyses, including EEG preprocessing, feature calculations, statistics, and classifications, were implemented using the MATLAB 2019b software.

## 3. Results

**Figure 1:**
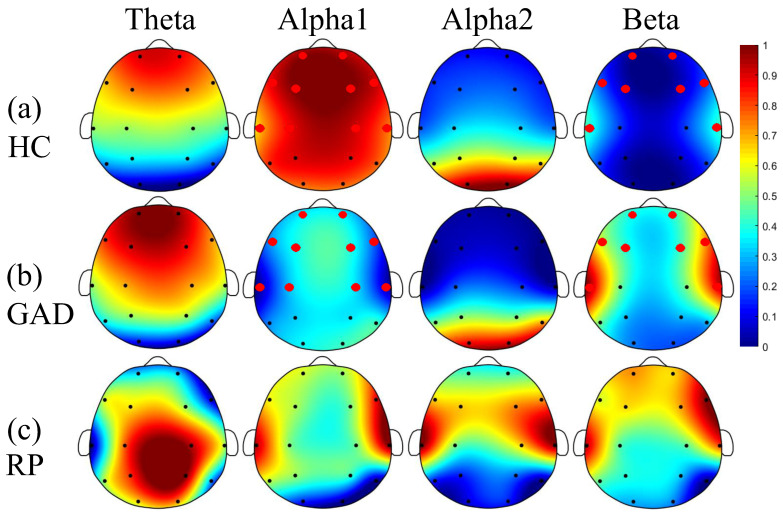
Brain topography of the PSD for the four EEG rhythms. Each value is the average of all subjects. The relative powers of HC (**a**) and GAD (**b**) have been normalized between 0 and 1 for the theta, alpha1, alpha2, and beta rhythms for the sake of better visualization, so they share the same color bar. The red dots represent these EEG channels have significant differences (*p* < 0.05). The subgraphs of (**c**) are the relative PSD (RP: |PSD_GAD_-PSD_HC_|/PSD_HC_) of GAD relative to HC for the four rhythms.

**Figure 2:**
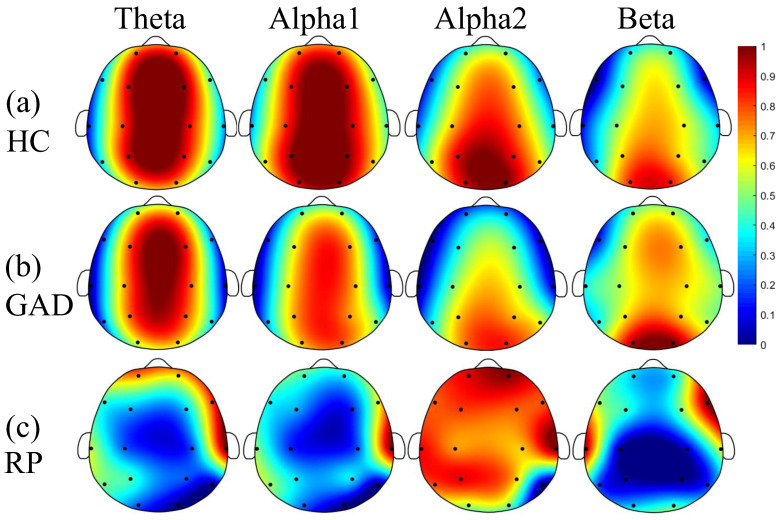
Brain topography of the FE for all EEG rhythms. Each value is the average of all subjects. The FE of HC (**a**) and GAD (**b**) have been normalized between 0 and 1 for the theta, alpha1, alpha2, and beta rhythms for the sake of better visualization, so they share the same color bar. All rhythms have no significant differences (*p* > 0.05). The subgraphs of (**c**) are the relative FE (RFE: |FE_GAD_–FE_HC_|/FE_HC_) of GAD relative to HC for the four rhythms.

Figure 1 and Figure 2 demonstrate the results of PSD and FE analysis between GAD and HC. Comparing Figure 1a with Figure 1b, it suggests that slow EEG rhythms (theta and alpha1) have lower relative power, and fast EEG rhythms (alpha2 and beta) have higher relative power in GAD than in HC. Meanwhile, only alpha1 rhythm in the frontal, temporal and central areas and beta rhythm in the frontal and temporal areas have significant statistical differences, and the most significant changes are distributed in the frontal and temporal regions (see the topography of alpha1 and beta rhythm in Figure 1c). In Figure 2a,b, the FE of theta, alpha1, and alpha2 rhythms in GAD exhibit a decreasing trend compared with HC. Meanwhile, beta rhythm shows a trend of increase; but all rhythms have no statistical differences.

**Figure 3:**
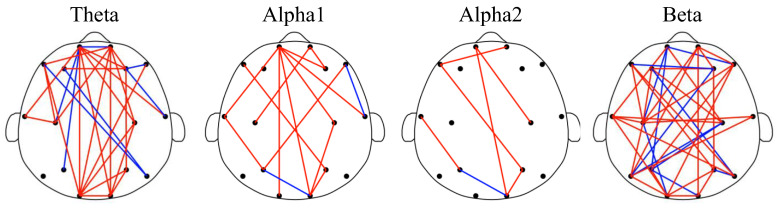
Brain functional network of theta, alpha1, alpha2, and beta rhythms. In the brain functional networks, the red edge means the PLI value of GAD is lower than that of HC. Meanwhile, the blue edge represents the PLI value of GAD is higher than that of HC.

Figure 3 shows the results of the FC analysis. In Figure 3, the FCs with statistical differences are demonstrated in the brain functional networks. Interestingly, 77% of the existed FCs (7/30 for theta, 2/15 for alpha1, 1/7 for alpha2, and 11/40 for beta) have lower values in GAD compared to HC. The topological distributions of the brain networks indicate that the FCs are mainly related to the frontal area and distributed between frontal and other areas. Specifically, the ratio of the FCs related to frontal area is 67% in total, and 24/30, 11/15, 4/7, and 23/40 for theta, alpha1, alpha2, and beta rhythms, respectively. To sum up, the results suggest the reduced long-range brain interactive activities between frontal and other areas among the patients with GAD.

In addition, three popular classifiers of SVM, RF, and BP_Bagging are applied for identifying GAD and HC with the aberrated EEG features (18 PSD features, 0 FE features, and 92 FC features). As shown in Table 2, the classification accuracies with these three classifiers are 97.83 ± 0.40%, 90.16 ± 0.92%, 95.51 ± 0.20%, respectively. We also calculate the accuracies of the four rhythms separately (theta, alpha1, alpha2, and beta rhythms contains 30, 25, 7, 48 EEG features, respectively). The highest accuracies for each rhythm are 70.92 ± 0.80%, 73.67%, 69.46%, and 96.49 ± 0.33%, respectively. Among these four EEG frequency bands, beta rhythm acquires the highest accuracy, and is very close to the results of the accuracy obtained with all features.

## 4. Discussion

In this resting-state EEG study, we provided a comprehensive analysis with three widely used types of EEG feature extraction strategies, which can decode the EEG information from three different dimensions, to reveal the neurophysiological alterations of GAD. The significant findings are as follows: first, beta rhythm emerged with significant changes both in PSD analysis and machine learning analysis, depicted by increased relative power in GAD compared to HC. Second, the statistics of FC values and distributions indicated that GAD has the reduced long-range brain interactive activities between frontal and other areas in all frequency bands. Third, the highest classification accuracy of 97.83 ± 0.40% for GAD detection was acquired with the extracted multidimensional EEG features, suggesting good performances of extracting aberrated EEG features. These findings are discussed in greater detail below.

### 4.1. Striking EEG Rhythm in GAD

Although numerous studies have used EEG rhythms as reliable characteristic patterns for GAD studies [19], our study further supports the important role of beta rhythm in GAD through different perspectives. On the one hand, beta rhythm had statistical differences between GAD and HC in PSD analysis. On the other hand, beta rhythm had the highest classification accuracy compared to theta, alpha1, and alpha2, which means the largest difference of beta rhythms were between GAD and HC than that of other rhythms. Although alpha1 rhythms also had statistically difference in the PSD analysis, the classification accuracy of alpha1 rhythms (73.67%) was much lower compared to beta rhythms (96.49 ± 0.33%). In fact, few previous studies reported the similar conclusion of the important role of beta rhythm in GAD. A direct reason may be the lack of multi-methodological comparative studies with EEG signals. To our knowledge, there is no study performed multidimensional feature extraction and machine learning on the EEG signals with GAD. More attention has been paid to the delta-beta cross-frequency correlation [22]. The striking beta rhythm can help to be better understanding the neuromechanism of GAD.

The increases of beta relative power and FE indicated that patients’ brain activities are often keeping them in a state of nervousness and neural disorganization. Previous studies have also widely reported the significant increase in beta power [19,33,34] and the similar distribution of the frontal region [35], which is inconsistent with our study. Beta rhythm is usually associated with the vigilant and excited state of the brain [22,36,37] and mediates higher cognitive functions [38] (for instance, ‘top-down’ modulation of brain processing [39]). By contrast, theta rhythm is linked with emotional influences on perception [38,40]. The decrease in slow EEG relative powers (theta and alpha1) and significant increase in fast EEG (beta) powers reflected negative emotions and significant activation of the brain [41]. Meanwhile, FE performs an improved evaluation of signal complexity and has been powerfully applied to EEG signals [42,43]. The slight increase in FE of beta rhythm indicated the increased complexity of EEG signals and prompted the brain neural disorganization. A similar study based on correlation dimension analysis also pointed out the increase complexity of the EEG signals with GAD [9]. One possible explanation may be the increased worry/internal cognitive processing of the GAD in the form of divergent negatively biased mind wandering (i.e., considering all possible outcomes leading to “catastrophic thinking”) during non-specific information processing [11]. To sum up, the significant changes in beta rhythm can help to well explain the neural mechanism of GAD, and provide basic theoretical supports for subsequent research on detection methods and neural marker identification.

This current study was also motivated by previous studies which have divided the EEG bands into narrower frequency bands [19]. Here, we firstly divided alpha rhythm into two sub-bands (alpha1 and alpha2) in GAD research, and obtained some meaningful results. That is, the relative power of alpha1 rhythm and alpha2 showed a different changing mode and statistical analysis results, suggesting the essential process in EEG-related GAD research to divide alpha frequency band into alpha1 and alpha2 sub-bands. Klimesch et al. have reported that narrower frequency bands can reduce the risk of offsetting or undetected frequency effects [44]. In summary, narrower band divisions can enhance the physiological significance of sub-bands, which is supported by the results of alpha1 and alpha2 relative powers.

### 4.2. Reduced FCs and Dominant Brain Areas

In this study, PLI was utilized to estimate brain FC, which can overcome the volume conduction problem during EEG acquisition [32]. Significantly, we observed the reduced weights of most FCs in GAD through statistical analysis, and these statistical FCs were significantly correlated with prefrontal area and mainly distributed between frontal and other brain areas. We can conclude that GAD has reduced FC between frontal areas and other brain regions.

To date, scant FC studies based on EEG signals have been performed on GAD [11]. As it is known, different subtypes of anxiety disorders exhibit different specific phenotypes [11,22]. Some relevant studies of other subtypes of anxiety disorder (such as panic disorder, social anxiety disorder, trait anxiety disorder) based on brain FC give our findings some support [2,45]. Panic disorder patients have lower weights of inter-hemispheric FCs in the frontal region and intra-hemispheric FCs in the bilateral temporal region [46]. Lower FC is associated with higher trait anxiety disorder [47], and dysregulated alpha connectivity is observed in trait anxiety disorder [48]. Conversely, an increase in oscillatory coherence of the theta rhythm indicated the higher connectivity in the social anxiety disorder compared to HC during resting-state EEG study [49]. Increasing evidence supports the notion that anxiety disorders are associated with abnormal communication between brain regions. Existed studies also proved that different subtypes of anxiety disorder are modulated by discrete neurobiological mechanisms.

### 4.3. Excellent Classification Performance for GAD Detection

In this study, we obtained excellent classification performance, with an accuracy of up to 97.83 ± 0.40% between GAD and HC groups, which is better than existing similar studies. Mokatren et al. obtained a highest accuracy of 92.19% with a convolutional neural network model and an 81.25% accuracy with a SVM classifier applied on social anxiety disorder with resting-state EEG dataset [26]. Park et al. reported their highest accuracy of 91.03% with elastic net classifier using resting-state EEG data [28]. Al-Ezzi et al. achieved the accuracies of 92.86%, 92.86%, 96.43%, and 89.29% for severe, moderate, mild anxiety and HC by using a deep learning model (convolutional neural network + long short-term memory) with task-state EEG data, respectively [27]. Moreover, lower classification accuracies were reported using other modalities data [50,51,52], such as an accuracy of 87.4% with Self-Rating Anxiety Scale questionnaires data [12], and an accuracy of 86% with language-based features [53]. The above studies give us the following insight: better performance with our proposed EEG feature extraction and selection than the results of other existing studies indicates the importance of the EEG feature extraction process in classification problems. Compared to Mokatren’s SVM result, we achieved much higher accuracy than Mokatren’s. This is most likely because only relative power and wavelet entropy were used for SVM in Mokatren’s study, without FC method and feature selection method.

### 4.4. Limitations

Although the current results may be intriguing, some limitations should be considered. Firstly, there were 45 patients with GAD and 36 HC volunteers enrolled in this study. This sample size is not big enough to draw definitive conclusions. Secondly, the participants were between 18 and 55 years old. A broader age range needs to be designed to obtain more comprehensive findings in future research. Thirdly, a 16 electrode EEG system was used for this study. Further research will focus on a high-density EEG system (for example, 64 electrodes) and compare the results with these findings. Fourthly, only one of the anxiety subtypes, GAD, was explored herein. Other anxiety disorder subtypes and deep learning models will be considered for the identifications among the subtypes. Finally, three widely used EEG assessments that have been proven in detecting mental disorders were adopted here for multidimensional EEG feature construction. Additional efforts are needed to exploit for the best combination of EEG measurements to replicate our findings and more importantly further improve the detection performance.

## 5. Conclusions

In this study, we developed an EEG feature extraction and selection framework for the automatic detection of GAD with resting-state EEG. By incorporating multidimensional EEG characteristics and statistical analysis, we found that individuals with GAD exhibited a significant increase in beta rhythm and a decrease in alpha1 rhythm of relative power, together with the reduced long-range brain interactive activities between frontal and other brain areas in all frequency bands, which can be served as the neural biomarkers of GAD. Furthermore, to the best of our knowledge, the highest classification performance with 97.83 ± 0.40% of accuracy, 97.55 ± 0.31% of sensitivity, 97.78 ± 0.36% of specificity, and 97.95 ± 0.17% of F1 was achieved, based on aberrated multidimensional EEG features. Overall, the subjective and electrophysiological findings reported herein have potential applications for the developments of electrophysiological neuromechanisms and automatic diagnosis of GAD.

## Figures and Tables

**Table 1:** Demographic and clinical characteristics of the participants.

Characteristics	GAD (*n* = 45)	HC (*n* = 36)	t	F	*p*-Value
Age (year)	22–55 (41.8 ± 9.4)	21–57 (36.9 ± 11.3)	1.99	3.96	0.06
Gender: male/female	13/32	11/25	-	-	-
Duration of illness (month)	1–48 (7.9 ± 7.6)	-	-	-	-
HAMA	27.1 ± 9.0	2.3 ± 0.9	16.63	222.18	1.14 × 10^−24^
HAMD-17	10.6 ± 6.0	2.4 ± 0.8	17.70	253.59	2.24 ×10^−26^

**Table 2:** Classification accuracies with different feature groups between GAD and HC.

Models	Index (%)	All	Theta	Alpha1	Alpha2	Beta
SVM	Accuracy	97.83 ± 0.40	70.92 ± 0.80	73.39 ± 0.56	63.13 ± 0.40	96.49 ± 0.33
	Sensitivity	97.55 ± 0.31	74.00 ± 0.80	75.91 ± 0.65	66.64 ± 1.03	96.83 ± 0.34
	Specificity	97.78 ± 0.36	66.12 ± 1.16	69.76 ± 0.15	56.89 ± 2.23	95.82 ± 0.44
	F1	97.95 ± 0.17	74.33 ± 0.55	76.91 ± 0.58	67.71 ± 0.49	96.83 ± 0.23
RF	Accuracy	90.16 ± 0.92	69.59 ± 0.69	73.67 ± 0.91	69.46 ± 0.44	88.76 ± 0.52
	Sensitivity	88.82 ± 1.08	70.16 ± 0.97	72.01 ± 0.70	68.49 ± 0.80	88.30 ± 0.98
	Specificity	91.69 ± 0.51	68.32 ± 0.46	77.90 ± 1.55	70.70 ± 0.98	89.71 ± 1.17
	F1	91.44 ± 0.65	75.13 ± 0.72	79.31 ± 0.77	75.78 ± 0.66	90.45 ± 0.18
BP_Bagging	Accuracy	95.51 ± 0.20	68.42 ± 0.80	71.37 ± 0.39	68.99 ± 0.33	93.41 ± 0.85
	Sensitivity	88.74 ± 0.88	73.45 ± 0.66	71.94 ± 0.53	66.56 ± 0.48	88.54 ± 0.52
	Specificity	91.98 ± 0.97	66.23 ± 1.34	78.26 ± 1.56	68.78 ± 1.47	89.74 ± 0.84
	F1	91.50 ± 0.38	72.22 ± 3.75	79.30 ± 0.42	73.87 ± 0.14	90.58 ± 0.56

## Data Availability

Not applicable.
